# Retraction: Mutations in the Fusion Protein Cleavage Site of Avian Paramyxovirus Serotype 4 Confer Increased Replication and Syncytium Formation *In Vitro* but Not Increased Replication and Pathogenicity in Chickens and Ducks

**DOI:** 10.1371/journal.pone.0244076

**Published:** 2020-12-14

**Authors:** The PLOS ONE Editors

After this article [1] was published, concerns were raised about results reported in Figs 3A and 5.

Specifically:

- The Mock-infected panel of Fig 3A in [1] appears similar to the Mock-infected panel of Fig 2A in [2]. The authors confirmed that they used the same image to represent the mock-control in both publications. They stated that the same DF1 cells were used as a control for both of the experiments, and that per their observations there is little difference in batch-to-batch results for mock-infected DF1 cells.
- The APMV-1 (BC) and rAPMV-4/ Fc SV panels of Fig 5 in [1] appear similar to the APMV-1 (BC) and APMV-8 panels, respectively, of Fig 3A in [3]. The authors confirmed the same APMV-1 (BC) image was used in both figures; they stated that the two experiments were conducted simultaneously and that the same APMV-1 control applied to both. Regarding the rAPMV-4/ Fc SV [2] and APMV-8 [3] panels, the authors commented that data reuse may have resulted from an error in figure assembly.

The authors noted that the original data underlying the results reported in this article are no longer available. There are fifteen instances where the Results section refers to data not shown, and the data underlying those statements are likewise not available.

This article was included in an investigation by the Department of Health and Human Services (HHS), which concluded there was evidence of research misconduct in the case of the first issue described above [4].

In light of the above concerns, the unavailability of the original data, and the outcome of the HHS investigation, the *PLOS ONE* Editors retract this article.

SKS agreed with retraction. HS agreed with retraction and apologized for the issues with the published article. PLC agreed with retraction, stands by the article’s findings, and apologized for the issues with the published article. S-HK did not agree with retraction and stands by the article’s findings. SX either could not be reached or did not respond directly.

## References

[1] Kim S-H, Xiao S, Shive H, Collins PL, Samal SK (2013) Mutations in the Fusion Protein Cleavage Site of Avian Paramyxovirus Serotype 4 Confer Increased Replication and Syncytium Formation *In Vitro* but Not Increased Replication and Pathogenicity in Chickens and Ducks. PLoS ONE 8(1): e50598 10.1371/journal.pone.0050598 23341874 PMC3544850

[2] Kim S-H, Wanasen N, Paldurai A, Xiao S, Collins PL, Samal SK (2013) Newcastle Disease Virus Fusion Protein Is the Major Contributor to Protective Immunity of Genotype-Matched Vaccine. PLoS ONE 8(8): e74022 10.1371/journal.pone.0074022 24015313 PMC3755997

[3] Kim S-H, Xiao S, Shive H, Collins PL, Samal SK (2012) Replication, Neurotropism, and Pathogenicity of Avian Paramyxovirus Serotypes 1–9 in Chickens and Ducks. PLoS ONE 7(4): e34927 10.1371/journal.pone.0034927 22558104 PMC3340391

[4] (2020) FR Doc. 2020–10253. Federal Register 85(93): 28643–28645. 32435075 PMC7220845

